# Alpha-1-antitrypsin functions as a protective factor in preeclampsia through activating Smad2 and inhibitor of DNA binding 4

**DOI:** 10.18632/oncotarget.22949

**Published:** 2017-12-05

**Authors:** Yaling Feng, Nan Wang, Jianjuan Xu, Jinfang Zou, Xi Liang, Huan Liu, Ying Chen

**Affiliations:** ^1^ Department of Obstetrics and Gynecology, Wuxi Maternal and Child Health Hospital Affiliated to Nanjing Medical University, Wuxi, Jiangsu 214002, PR China; ^2^ Department of Obstetrics, The Third Xiangya Hospital of Central South University, Changsha, Hunan 410013, PR China; ^3^ Central Lab, Wuxi Maternal and Child Health Hospital Affiliated to Nanjing Medical University, Wuxi, Jiangsu 214002, PR China

**Keywords:** pre-eclampsia, alpha-1-antitrypsin, Smad, inhibitor of DNA binding, whole-exome sequencing

## Abstract

Pre-eclampsia (PE) is one of the most common reason for high morbidity and mortality of maternal and prenatal infants. Production from oxidative stress results in maternal ROS system and anti-oxidation defense system imbalance to promote tissue ischemia and hypoxia, and ultimately impairs the maternal organs and placenta. Our previous study showed that exogenous Alpha-1-antitrypsin (AAT) and overexpression of AAT in umbilical vein cell (HUVEC) hypoxia-reoxygenation model could increase the activity of antioxidant enzymes, and played a protective role in preeclampsia animal model. In this study, we aim to investigate the underlying mechanism by which AAT prevents PE progress. Whole-exome sequencing was performed to screen the genes altered by AAT. We found that AAT knockdown altered the expression of Smad family and Id family genes, and further demonstrated that AAT positively regulated Id4 expression through activating Smad2. Reduced Id4 expression and Smad2 phosphorylation were observed in preeclampsia animal model, which was also confirmed in human placenta tissues. In addition, AAT protected HUVEC cells from hypoxia/reoxygenation injury and relieved preeclampsia symptoms through Smad2/Id4 axis. Our data illustrate AAT/Smad2/Id4 axis is an important mediator of placenta and vascular function during pregnancy. These findings provide insights into events governing pregnancy-associated disorders, such as preeclampsia.

## INTRODUCTION

Pre-eclampsia (PE), a pregnancy-specific disease accounting for pregnancy complications of 5% to 7%, is one of the most common reason for high morbidity and mortality of maternal and perinatal infants [1]. Oxidative stress theory considers that the placental excess oxidative stress can lead to weaken of trophoblast trophoblast cell migration and invasion, and then cause uterine spiral artery recanalization disorder and shallow placental implantation. And deposition of oxidative stress products ROS in the blood vessels results in maternal ROS system and anti-oxidation defense system imbalance to promote vascular endothelial cell injury, systemic arteriolar spasm, tissue ischemia and hypoxia, and ultimately impairs the maternal organs and placenta [2].

Our previous study showed that exogenous Alpha-1-antitrypsin (AAT) and overexpression of AAT in umbilical vein cell (HUVEC) hypoxia-reoxygenation model could increase the activity of antioxidant enzymes and decrease the apoptosis [3]. It is speculated that AAT can protect endothelial from oxidative stress injury and is involved in the development of preeclampsia.

Inhibitor of DNA binding (ID) family protein is a class of transcription factors, including ID1 / 2/3/4. They play an important role in the regulation of endothelial cell cycle, proliferation, migration and angiogenesis and invasion [4]. It has been found that activation of bone morphogenetic protein (BMP) receptors can lead to phosphorylation of Smad5 and promote the migration of endothelial cells, while ID1 is identified as a target gene for BMP/Smad; interference Id1 can block BMP-mediated endothelial cell migration [5, 6]. BMP receptor mutations are often found in hereditary pulmonary hypertension, which can lead to a decrease in Smad1/5-driven Id1 expression [7]. Prostaglandin can enhance the expression of Smad1/5 phosphorylation and Id1 induced by BMP, and its mechanism is related to inhibitory Smad6. In addition, prostacyclin can inhibit the proliferation of smooth muscle cells, thereby preventing the progression of pulmonary hypertension and increase Smad1/5 phosphorylation and Id1 expression [8]. In the inflammatory response, the expression of Id1 in endothelial cells will increase in adaptive expression. And the study also found that TGFβ/Smad pathway also plays an important role in vascular endothelial cells, inhibition of Smad6/7 can antagonize TGFβ-induced pulmonary artery endothelial cell permeability disorder [9]. It has been found that ID2 can regulate the differentiation, migration and invasion of trophoblast cells [10]. Moreover, in the early stage of eclampsia, the expression of Smad2 phosphorylation and Smad7 in placental tissue is increased and plays an important role in the proliferation and differentiation of trophoblast cells [11].

These results suggest that Smad family proteins and ID family proteins play an important role in the proliferation, migration, angiogenesis and permeability of vascular endothelial cells, and may play a role in preeclampsia by regulating the inflammatory response and endothelial cell function. Thus, we propose that AAT positively regulate Ids expression through Smads to play a role in endothelial cells or trophoblast proliferation, migration, angiogenesis and permeability, finally contribute to prevention of preeclampsia progression.

## RESULTS

### The gene alterations resulted from AAT knockdown

**Figure 1:**
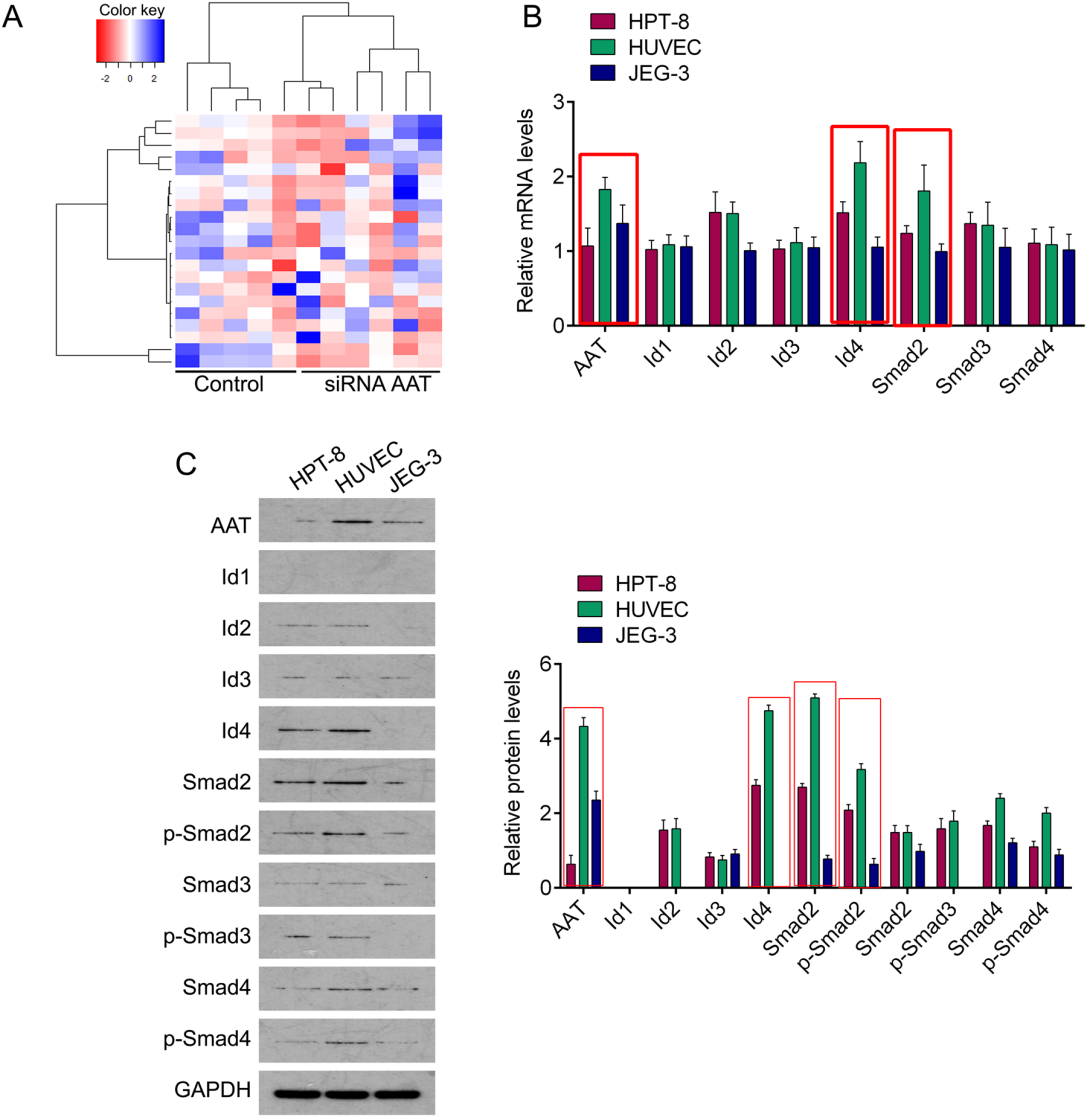
The expression profile of AAT knockdown related genes **(A)** Heat-map of genes altered by AAT knockdown. **(B)** QPCR was performed to measure the mRNA expression of AAT, Id1, Id2, Id3, Id4, Smad2, Smad3 and Smad4 in HPT-8, HUVEC and JEG-3 cells. **(C)** Western blot was performed to measure the protein expression of AAT, Id1, Id2, Id3, Id4, Smad2, Smad3 and Smad4 in HPT-8, HUVEC and JEG-3 cells. Right panel, Quantification of the bands.

Whole-exome sequencing in AAT knockdown cells and control cells was performed. Each sample obtained approximately 3.32~4.82 Gb of cleaned sequencing data. As shown in Table 1, AAT downregulation resulted in down-regulation or up-regulation of genes expression. The top ten of upregulated genes included ANKRD36B, MMP1, NBN, BIRC3, GOLGA8B, LOC100508892, HMMR, PUS7L, FAM111B and STAG2SYCP1. And the top ten of downregulated genes included ID2, ID4, SMAD2, PPIA, EGR4, ST6GALNAC2, ID1, SMAD4, GPR56 and IER5L (Table 1). Among them, the Id family proteins and Smad family proteins were frequently observed in the downregulated genes. Especially, the fold changes of ID2, ID4 and SMAD2 were more than 6 (Table 1). In order to explore the possible mechanism by which AAT influences the progress of preeclampsia, we especially concerned with the Id family and Smad family proteins. We then performed qPCR and western blot in human umbilical vein endothelial cells (HUVEC), trophoblast cell (hpt-8) and chorionic cancer cells (jeg-3) cells to confirm the expression profile of these genes. We found that AAT mRNA expressed in all the three cell lines with highest in HPT-8 cells, and decreased in JEG-3 cells. The expressive profiles of Id4 and Smad2 were similar to AAT. The expression of other genes was slightly fluctuated (Figure 1B). These phenomena were also confirmed in protein levels evaluated by western blot (Figure 1C), suggesting that AAT may through regulating Id4 and Smad2 to contribute to preeclampsia.

**Table 1:** The top ten upregulated and downregulated genes in cells knocked down AAT

Genes	Fold change (siRNA AAT vs. control)
**Upregulated genes**	
ANKRD36B	7.42
MMP1	7.34
NBN	6.39
BIRC3	6.21
GOLGA8B	5.94
LOC100508892	5.94
HMMR	5.84
PUS7L	5.53
FAM111B	5.33
STAG2	5.08
**Downregulated genes**	
ID4	-11.47
ID2	-7.67
SMAD2	-6.17
PPIA	-3.14
EGR4	-3.12
ST6GALNAC2	-2.99
ID1	-2.98
SMAD4	-2.90
GPR56	-2.87
IER5L	-2.81

### AAT enhances Id4 expression through activating Smad2

**Figure 2:**
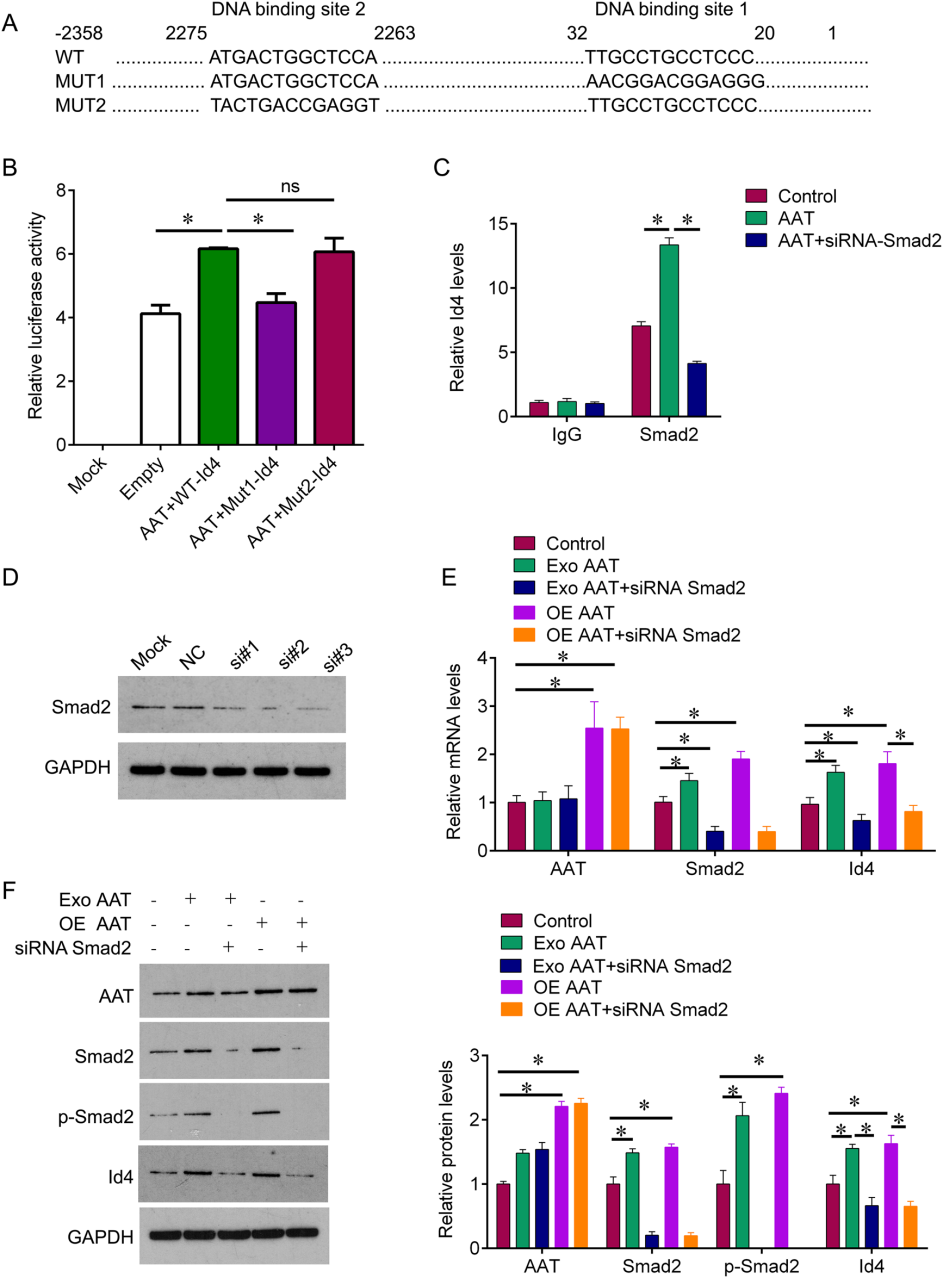
AAT enhances Id4 expression through activating Smad2 **(A)** The predicted binding sites of Smad2 on wild type Id4 promoter, and the mutant type of Id4 promoter. **(B)** The cells were treated with AAT together with wild type Id4 promoter or the mutant type, dual luciferase reporter gene assay was used to measure the activity of promoter. The untreated cells were used as mock control, the cells treated with empty lentivirus were used as negative control. **(C)** Chip was performed to verify the interaction of Smad2 and Id4. IgG pull down was used as negative control. **(D)** Western blot was performed to measure the protein expression of Smad2 in HUVEC cells. Si#1, si#2, si#3, the three Smad2 siRNA sequences. **(E)** QPCR was performed to measure the mRNA expression of AAT, Id4 and Smad2 in HUVEC cells after indicated treatment. **(F)** Western blot was performed to measure the protein expression of AAT, Id4, Smad2 and Smad2 phosphorylation in HUVEC cells after indicated treatment. Exo, exogenous; OE, overexpression. ^*^p<0.05.

To investigate the regulatory relationship, we next predicted whether the transcript factor Smad2 could bind to Id4. Using the bioinformatic tool in JASPAR database, we found that two potential binding sites of Smad2 were found in the promoter of Id4 (Figure 2A). To confirm this prediction, we constructed the plasmids that containing wild type promoter of Id4 and the mutant binding sites to perform the dual luciferase reporter gene assay. The results showed that AAT induced significant increase of activity of wild type Id4 promoter compared with negative control. When the binding site 1 was mutant, the activity of Id4 promoter was significantly decreased; in contrast, the mutation of binding site 2 did not alter the activity of Id4 promoter (Figure 2B). In addition, Chip results also showed that AAT treatment increased the expression of Id4 that binding to Smad2, and knockdown of Smad2 by siRNA could reduce AAT-mediated expression of Id4 (Figure 2C). Furthermore, we knocked down the Smad2 expression by siRNA; and one of most effective siRNA sequence si#3 was used for following experiments (Figure 2D). we confirmed that exogenous AAT and overexpression of AAT could induce the expression of Smad2 and Id4 at mRNA and protein levels, whereas knockdown of Smad2 abolished exogenous AAT and overexpression of AAT-mediated enhancement of Id4 (Figure 2E). These results demonstrated that Smad2 binds to Id4 promoter and AAT stimulates Id4 expression through Smad2.

### Cytoprotective role of AAT in I/R cell model was reversed by Id4 knockdown

**Figure 3:**
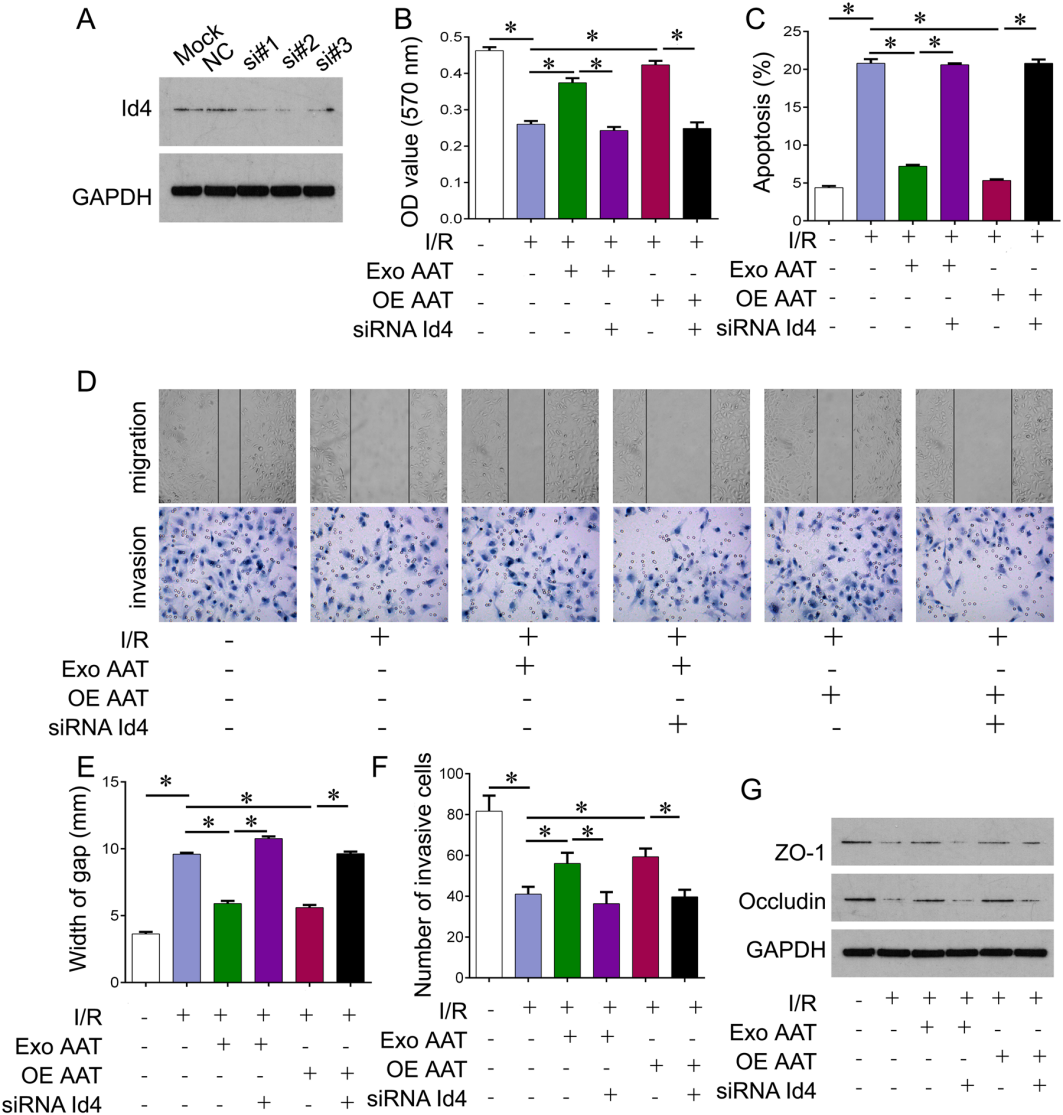
Cytoprotective role of AAT in I/R cell model was reversed by Id4 knockdown **(A)** Western blot was performed to measure the protein expression of Id4 in HUVEC cells. Si#1, si#2, si#3, the three Id4 siRNA sequences. **(B)** CCK-8 was performed to measure the cell viability of HUVEC cells after indicated treatment. **(C)** Flow cytometry was performed to measure the aopotosis of HUVEC cells after indicated treatment. **(D)** Scratch (upper) and transwell (lower) assay were performed to evaluate migration and invasion ability of trophoblast cell. **(E)** Measurement of width gap for scratch assay in (D). **(F)** Measurement of invasive cells for transwell assay in (D). **(G)** Western blot was performed to measure the cell membrane permeability related genes, ZO-1 and Occludin protein expression in HUVEC cells after indicated treatment. Exo, exogenous; OE, overexpression. ^*^p<0.05.

Our previous study has shown that AAT plays cytoprotective role in I/R model. Here we investigated whether AAT protected the cells from I/R injury through Id4. We knocked down Id4 by siRNA; and one of most effective siRNA sequence si#2 was used for following experiments (Figure 3A). We treated the I/R model cells with exogenous AAT or AAT expressed lentivirus, or together with Id4 siRNA. I/R resulted in decreased cell viability compared with control cells. Exogenous AAT and overexpression of AAT protected cells from I/R injury, whereas knockdown of Id4 by siRNA reversed this protection (Figure 3B). We also confirmed these phenomena by apoptosis analysis using flow cytometry assay (Figure 3C). In addition, we performed transwell and scratch assay to evaluate the role of AAT on migration and invasion of trophoblast cells. We observed that I/R challenge significantly inhibited trophoblast cell migration and invasion compared with control. And we found that exogenous AAT and overexpression of AAT promoted trophoblast cells migration and invasion, whereas knockdown of Id4 by siRNA reversed this promotion (revised Figure 3D-3F). We also examined two markers of cell membrane permeability, ZO-1 and Occludin. I/R induced significant decrease of ZO-1 and Occludin, while exogenous AAT and overexpression of AAT restored the expression of ZO-1 and Occludin. However, knockdown of Id4 by siRNA reversed this restoration (Figure 3G).

### AAT reliefs preeclampsia symptoms through Id4

**Figure 4:**
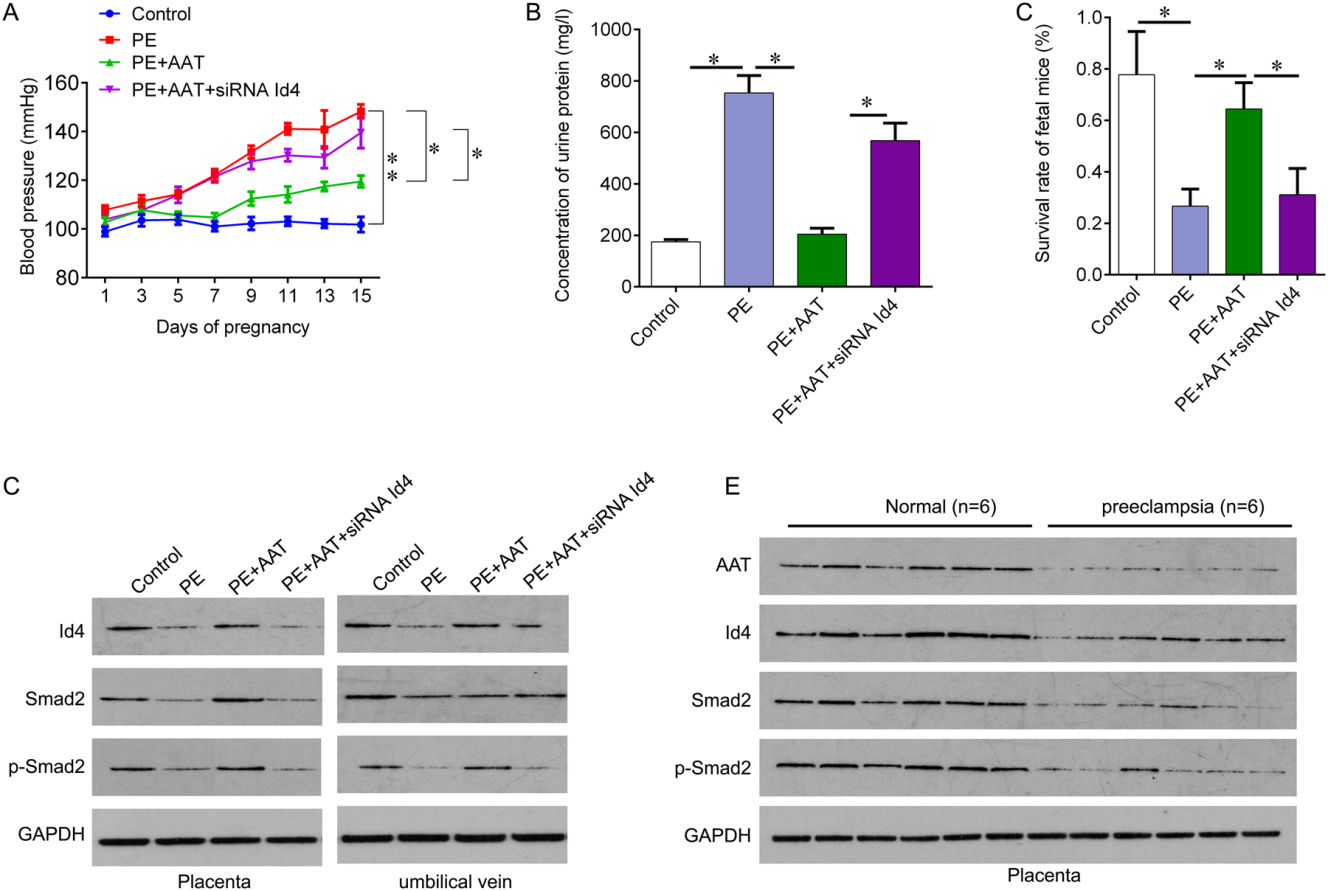
AAT reliefs preeclampsia symptoms through Id4 **(A)** Blood pressure in preeclampsia animal model after indicated treatment. **(B)** Proteinuria ELISA Kit was used to measure the concentration of urine protein on day 16 of gestation after indicated treatment. **(C)** Survival rate of fetal mice was calculated after delivery. **(D)** Western blot was performed to measure the protein expression of AAT, Id4, Smad2 and Smad2 phosphorylation in placenta and umbilical vein tissues obtained from animal model after indicated treatment. **(E)** Western blot was performed to measure the protein expression of AAT, Id4, Smad2 and Smad2 phosphorylation in placenta tissues obtained from health and preeclampsia subjects (N=6). PE, preeclampsia; Exo, exogenous; OE, overexpression. ^*^p<0.05, ^**^p<0.01.

We next investigate the role of AAT in a preeclampsia animal model. The animal model mimics the hypertension during pregnancy by injecting sFlt-1. The mice were injected with sFlt-1 alone or together with AAT (50mg/kg), or together with Id4 siRNA lentivirus. Seven days of sFlT-1 injection resulted in significant increase in blood pressure compared with the control. And AAT injection decreased the blood pressure induced by sFlt-1, which was attenuated by Id4 siRNA (Figure 4A). The increased urine protein induced by sFlt-1 also reduced by exogenous AAT treatment; Id4 knockdown abolished AAT-decreased concentration of urine protein (Figure 4B). Importantly, exogenous AAT treatment significantly increased the survival rate of fetal mice compared with PE group (Figure 4C). We then confirmed the expression of Id4 and Smad2 in placenta and umbilical vein tissues from model mice. Preeclampsia resulted in inactivation of Id4 and Smad2, which was re-activated by AAT (Figure 4D). In addition, we collected six normal placenta and preeclampsia placenta tissues from human to examine the expression of AAT, Id4 and Smad2. We also found that the expression of AAT, Id4 and Smad2 was significantly decreased in preeclampsia tissues compared with normal control (Figure 4E), confirming our results *in vitro* and animal model.

## DISCUSSION

In present study, we found that AAT (Alpha-1-antitrypsin) knockdown altered the expression of Smad family and Id family genes, and further demonstrated that AAT positively regulated Id4 expression through activating Smad2. In addition, AAT protected HUVEC cells from hypoxia/reoxygenation injury and relieved preeclampsia symptoms through Smad2/Id4 axis.

We previously constructed two-dimensional electrophoresis maps of decidual tissues to identify the differentially expressed proteins. It was revealed that AAT was highly expressed in normal full-term pregnancy, and lowly expressed in early-onset severe preeclampsia and late-onset severe preeclampsia [12]. In a prospective case-control study, Twina G et al also showed that AAT levels were lower in the preeclampsia group compared to healthy group, and correlated with protease inhibitory capacity [13]. Wen Q et al screened a 19-peptide panel including AAT to discriminate preeclampsia from confounding chronic hypertension with high specificity and sensitivity [14]. Recently, proteomic profiling also revealed AAT as preeclampsia-related serum proteins in pregnant women [15, 16]. In addition to serum, urine AAT may potentially serve as early indicators of preeclampsia [17]. These findings showed association between lower AAT levels and early or late-onset preeclampsia. Our previous studies demonstrated that exogenous AAT injection increased the antioxidants and suppresses oxidative stress, and subsequent prevention of preeclampsia development through inhibiting STAT1/ p38MAPK signaling [18, 19], suggesting that AAT would become a potential strategy for preeclampsia therapy.

Thus, in recent study we further explored the mechanism by which AAT targeted to contribute to preeclampsia progress. We here identified Smad2 and Id4 as two targets of AAT. Smad2 bound to the promoter of Id4 and activated it. Both exogenous AAT and overexpression AAT could increase the expression of Smad2 and Id4, while knockdown Smad2 by siRNA could abolish AAT-mediated increase of Id4. Thus, AAT positively regulated Id4 through increasing Smad2. Id proteins belong to the helix-loop-helix group of transcription factors and regulate cell differentiation and proliferation. They play important roles in cardiogenesis and formation of the vasculature. Id gene expression was dysregulated in heritable pulmonary arterial hypertension (HPAH) patients [20]. And previous studies showed the interaction between bone morphogenetic proteins and other growth factors or cytokines regulates Id gene expression [21]. Id-2 was downregulated at the mRNA and protein levels as the cytotrophoblast differentiated, but was maintained in placentas from women with preeclampsia, or in cells grown under hypoxic conditions in culture. And enhanced Id2 expression inhibited the cell invasion and migration, suggesting that Id-2 plays important roles in human cytotrophoblast development [10]. Id2 overexpression and knockdown analyses also indicated that Id2 mediated TGF-β-induced morphological differentiation of trophoblast cells [22]. SOCS box-containing 4 could degrade Id2 and mediate vascular differentiation in the placenta, inducing pathology that phenocopies human pre-eclampsia, including hypertension and proteinuria in late-stage pregnant females [23]. Here, we identified another Id protein Id4 was dysregulated in hypoxia/reoxygenation injury cells and preeclampsia mice placenta and umbilical vein tissues. AAT protected the cells from hypoxia/reoxygenation injury and prevented preeclampsia progress through Id4 that activated by Smad2. It was reported that constitutively expressing Smad7 could elevate endogenous Id2, Id3 and Id4 expression, which play a role in melanomagenesis. These Ids could suppress cyclin-dependent kinase inhibitors, and re-upregulate invasion and metastasis-related genes matrix metalloproteinase 2 (MMP2), MMP9, CXCR4 and osteopontin [24]. Yang J et al found that iloprost alone induced Id1 expression in human pulmonary artery smooth muscle cells independent of Smad1/5 activation, and enhanced BMP-induced phosphorylation of Smad1/5 and Id1 expression in a cAMP-dependent manner. They also confirmed that treprostinil inhibited smooth muscle cell proliferation and prevented progression of PAH while enhancing Smad1/5 phosphorylation and Id1 gene expression [7]. In present study, we demonstrated that AAT prevented progression of preeclampsia by enhancing Smad2 phosphorylation and Id4 gene expression. Reduced Id4 expression and Smad2 phosphorylation were observed in preeclampsia animal model, which was also confirmed in human placenta tissues. Our data illustrate AAT/Smad2/Id4 axis is an important mediator of placenta and vascular function during pregnancy. These findings provide insights into events governing pregnancy-associated disorders, such as preeclampsia.

## MATERIALS AND METHODS

### Cell lines

Human umbilical vein endothelial cells (HUVEC), trophoblast cell (hpt-8) and chorionic cancer cells (jeg-3) cells were obtained from Cell Bank of Chinese Academy of Sciences (Shanghai, China). The cells were grown in DMEM/F-12 (Gibco, Grand Island, NY, USA) supplemented with 10% fetal bovine serum (Gibco) at 37^°^C in 5% CO_2_.

### Lentivirus infection

To knock down AAT, Id4 and Smad2, the lentivirus containing AAT siRNA, Id4 or Smad2 siRNA (Genepharma, Shanghai, China) were constructed. Three siRNA sequences were constructed for Id4 and Smad2. The lentivirus (10^9^ U/ml) were used to infect cells or inject into animals. The cells infected with empty lentivirus were used as negative control. To overexpress AAT, the lentivirus containing AAT expressed plasmid (GeneCopoeia, Guangzhou, China) were constructed and used to infect cells. Cells were plated in 6-well clusters or 96-well plates and infected for 48 h for further assays or protein extraction.

### Whole-exome sequencing

To reveal the potential mechanisms how AAT relieves the symptoms of preeclampsia, we performed the whole exome sequencing (WES) analysis for screening the target genes. AAT was knocked down in HUVEC cells by siRNA. Genomic DNA samples were extracted from the cells knocked down AAT and negative control cells using TIANamp Genomic DNA Kits (Qiagen, Valencia, CA, USA). After evaluating DNA quality and quantity, 2 μg DNA was used for generating the sequencing library using the Agilent SureSelect Library Prep Kit according to the manufacturer's protocol. The SeqCap EZ Human Exome Library v3.0 (Nimblegen) were used to capture and enrich exome regions, which then were subjected to high-throughput sequencing using an Illumina HiSeq 2000/2500 platform to generate 101 bp paired-end reads.

### Quantitative real-time PCR analysis

Total RNA was extracted from cells using Trizol reagent (Invitrogen, CA, USA). RevertAid™ First Strand cDNA Synthesis kit (Thermo Fisher Scientific, Inc.) was used to reverse transcribe the mRNA to cDNA according to the manufacturer's protocol. Briefly, 0.5 ng of the template RNA, 1 μl Oligo(dT)18 Primer and 12 μl RNase free water were mixed, and incubated at 65°C for 5 min, then cooled down on ice. The mixture was added to Reaction Buffer (4 μl), RiboLock RNase inhibitor(1 μl), 10 mM dNTP mix (2 μl) and RevertAid Reverse Transcriptase(1 μl), and incubated at 42°C for 1 h. Finally, the mixture was heated to 70°C for 5 min to obtain the cDNA. The expression of AAT, Id1, Id2, Id3, Id4, Smad2, Smad3 and Smad4 was measured by SYBR green qPCR assay (Bio-Rad, USA) according to manufacturers’ instructions. Expression of β-actin was used as an endogenous control. The primers were used as followings: AAT, sense, 5’-TACCGACTGTTTCCCACACA-3’, anti-sense, 5’- TCAGAGAGGTTTGGCGACTT-3’; Id1, sense, 5’- CGGATCTGAGGGAGAACAAG-3’, anti-sense, 5’- CTGAGAAGCACCAAACGTGA-3’; Id2, sense, 5’- CCCAGAACAAGAAGGTGAGC -3’, anti-sense, 5’- AATTCAGAAGCCTGCAAGGA -3’; Id3, sense, 5’- GGAGCTTTTGCCACTGACTC -3’, anti-sense, 5’- CTTCAGGCCACAAGTTCACA -3’; Id4, sense, 5’- ATGGGATGAGGAAATGCTTG -3’, anti-sense, 5’- TTGGAGGAAGGAAAGCAGAA -3’; Smad2, sense, 5’- GTTCCTGCCTTTGCTGAGAC -3’, anti-sense, 5’- TTCTCTTTGCCAGGAATGCT -3’; Smad3, sense, 5’- TGCTGGTGACTGGATAGCAG -3’, anti-sense, 5’- CTCCTTGGAAGGTGCTGAAG -3’; Smad4, sense, 5’- CCCAGGATCAGTAGGTGGAA -3’, anti-sense, 5’- AAGGTTGTGGGTCTGCAATC -3’; β-actin, sense, AGGGGCCGGACTCGTCATACT, anti-sense, GGCGGCACCACCATGTACCCT. QPCR was performed at the condition: 95.0 °C for 5 min, and 39 circles of 94.0 °C for 20s and 61 °C for 20 s. Data were processed using 2^-ΔΔCT^ method.

### Western blotting

Proteins were extracted from indicated cells by RIPA buffer containing protease inhibitor PMSF (1%). The protein concentrations were determined by BCA Protein Assay Kit (Beyotime biotech Co. Ltd., Hangzhou, China) according to the manufacturers’ instructions. Sixty microgram protein was added on 10% SDS-PAGE and separated and transferred onto polyvinylidine difluoride (PVDF) membranes (Immobilon, Millipore) using Mini-PROTEAN® Tetra Cell Systems (Bio-Rad). Membranes were incubated with anti-AAT (mouse monoclonal antibody, 1:200, cat no. ab9399, Abcam), anti-Id1 (mouse monoclonal antibody, 1:1000, cat no. ab168256, Abcam), anti-Id2 (mouse monoclonal antibody, 1:500, cat no. ab166708, Abcam), anti-Id3 (mouse monoclonal antibody, 1:500, cat no. ab55269, Abcam), anti-Id4 (rabbit monoclonal antibody, 1:500, cat no. ab49261, Abcam), anti-Smad2 (rabbit monoclonal antibody, 1:2000, cat no. ab40855, Abcam), anti-Smad2 (phospho S467) (rabbit monoclonal antibody, 1:1000, cat no. ab53100, Abcam), anti-Smad3 (rabbit monoclonal antibody, 1:3000, cat no. ab28379, Abcam), anti-Smad3(phospho S423, S425) (rabbit monoclonal antibody, 1:2000, cat no. ab52903, Abcam), anti-Smad4 (rabbit monoclonal antibody, 1:2000, cat no. ab191026, Abcam), anti-Smad4(phospho T277) (rabbit monoclonal antibody, 1:2000, cat no. PAB1965, Abcam), anti-ZO-1 (rabbit monoclonal antibody, 1:500, cat no. ab96594, Abcam), anti-occludin (rabbit monoclonal antibody, 1:10000, cat no. ab167161, Abcam), or anti-GAPDH (mouse monoclonal antibody, 1:500, cat no. SC-365062, Santa Cruze) at 4°C overnight. Signals were visualized using ECL Substrates (Millipore, MA, USA). The IOD (integrated optical density) for each band was analyzed by Gel pro4.0 software.

### Cell viability assay

Cell viability was detected using CCK-8 Kit (Beyotime biotech Co. Ltd., Hangzhou, China). CCK-8 solution (10 μl) was added to each well of 96-well plates with the same amount of culture fluid. CCK-8 solution without cells was used as blank control. Optical density was determined at the time points on a microtiter plate reader at 570 nm.

### Flow cytometric analysis of apoptosis

eBioscience^™^ Annexin V Apoptosis Detection Kit (cat no. 88-8102-72, ThermoFisher Scientific, USA) was used for analysis of apoptosis according to the manufacturer's instructions. After indicated treatment, the cells were trypsinized, collected, and resuspended. About 2×10^5^ cells were harvested and washed twice with cold phosphate buffer saline (PBS), then resuspended in 500 μl binding buffer. 10μl Annexin V-FITC and 10 μl Propidium Iodide were added to the solution and mixed well. After 15 min incubation, the cells were analyzed using flow cytometric analysis (BD Biosciences, San Jose, CA).

### Luciferase reporter assay

The Id4 promoter region (2358 bp) and its mutant type (Figure 2A) was synthesized and inserted into a pGL3-basic vector (Promega, Madison, WI, USA), and then verified by DNA sequencing. Dual Luciferase Reporter Gene Assay Kit (cat no. GM-040502B, Genomeditech, Shanghai, China) was used to assess luciferase activities following manufacturer's protocol. The cells were plated in 96-well clusters, then cotransfected with 100 ng pGL3-basic vector or WT-Id4, Mut-1-Id4 or Mut-2-Id4 together with AAT or negative control. At 48 h after transfection, luciferase activity was detected and normalized to Renilla activity.

### Chromatin immunoprecipitation (ChIP) assay

The Pierce^™^ Agarose ChIP Kit (cat no. 26156, Thermo Scientific, USA) was used to conduct the ChIP assays in accordance with manufacturer's protocol, the HUVEC cells were fixed with 4% paraformaldehyde and incubated with glycine for 10 min to generate DNA–protein cross-links. Then, the cells were lysed with Cell Lysis Buffer and Nuclear Lysis Buffer and sonicated to generate chromatin fragments. Next, the lysates were immunoprecipitated with Magnetic Protein A Beads conjugated with Smad2-specific antibodies (Abcam), or IgG as a control. Finally, the precipitated DNA was analyzed by qRT-PCR. The primers specific for Id4 promoter were used as following: Smad2, sense, 5’- TCCGAAGGGAGTGACTAGGA -3’, anti-sense, 5’- CCGAGCCCAACAATTGAC -3’; And the primers for GAPDH were as sense, TACTAGCGGTTTTACGGGCG, anti-sense, TCGAACAGGAGGAGCAGAGAGC GA.

### Hypoxia/reoxygenation model

The hypoxia/reoxygenation model in HUVEC cells was established as we previously described [3]. For hypoxia, the cells were cultured in glucose and serum free media in normal incubator for 30 minutes, the cells then were exposed to hypoxia (1% O_2_) in an AW200SG hypoxic workstation (ELECTROTEK, UK) using a continuous flow of a humidified mixture of 1% O_2_, 5% CO_2_, and 94% N_2_ at 37°C for 4 h. For reoxygenation, after hypoxia, the cells were returned to culture in normal incubator with 20% O_2_, 5% CO_2_, and 75% N_2_ at 37°C for 18 h.

### Cell migration assay

Trophoblast cells after indicated treatments were cultured to full confluence. Wounds were created by 20 μl pipette tip. The cells were washed with serum-free medium for three times, and then incubated in DMEM added with 10% FBS for 24 h at 37°C. After that, the cells were observed and captured under an inverted microscope (Olympus, Japan). The width of gap was measured.

### Cell invasion assay

After indicated treatments, trophoblast cells suspension containing 5×10^5^ cells/ml was prepared in serum-free DMEM, and 300 μl of cell suspension was added into the upper chamber of the transwell chambers pre-coated with Matrigel (BD Pharmingen, San Diego, CA, USA). Then, 500 μl of DMEM added with 10% FBS was added into the lower chamber. After incubation for at 37°C 24 h, the invade cells were wiped out. The filters were fixed in 90% alcohol and stained by 0.1% crystal violet. Cell through the pores were observed and counted under an inverted microscope (Olympus, Japan).

### Preeclampsia model preparation

The female C57/BL6N mice were obtained from Hunan SJA Co. (Changsha, China). Animals were housed in a temperature-controlled room (23°C) with a 12:12 light: dark cycle. The experimental animals in this study were approved by the Institutional Animal Care and Research Advisory Committee of Nanjing Medical University. The preeclampsia model was prepared as previously described [25]. sFlt-1 (Recombinant mouse VEGF R1/Flt-1 Fc Chimera, Cat. No. 7756-FL-050, R&D Systems) was infused at a rate of 3.7 μg/kg/day for 6 days (in sterile saline) beginning on day 1 of gestation via miniosmotic pump (model 2001; Alzet Scientific Corporation, Palto Alto, CA) into normal pregnant rats. Normal pregnant/control groups (n=8) were fitted with a vehicle filled mini-osmotic pump. The preeclampsia mice were randomly divided into three groups each for 8 animals: PE+saline, PE+AAT, and PE+AAT+Id4 siRNA. Lentivirus Id4 siRNA (2 × 10^9^ titer units (TU)/ml, GeneChem Corporation, Shanghai, China) were used to knock down the expression of Id4 in PE mice. AAT solution (50 mg/kg/day for 6 days starting on day 13 of gestation, n=8) and lentiviral suspension (100 μl/day for 6 days starting on day 13 of gestation, n=8) were injected into tail veins. The preeclampsia mice treated with saline (n=8) were used as control.

### Blood pressure measurement and tissues collection

Non-invasive tail-cuff BP measurements were carried out in conscious mice using the non-invasive automated sphygmomanometer (BP-98A, Softron, Beijing, China). The mice were placed in a 38 degree incubator and warmed for 15 minutes to stabilize the mood and to expand the local vessels sufficiently, and then the pressurized sensor was placed in the tail. Mice blood pressure was measured after a few seconds of sobriety and tranquility. Each mouse was continuously measured 5 times and the average was taken as the final result. BP measurements were performed at the same time to ensure that blood pressure is relatively stable.

The proteinuria was measured on day 16 of gestation by using Proteinuria ELISA Kit (Abbexa, Cambridge, United Kingdom) according to manufacturer's instructions. After delivery, the survival rate of fetal was calculated as the number of live fetal divided by total number of fetal. And then the animals were euthanized under the anesthesia by overdose chloral hydrate. The peripheral and cord blood were collected for further analysis. And placentas and umbilical vessel were collected for western blot analysis.

### Statistical analysis

The SPSS Statistics 17.0 package was used to analyze data. All data from 3 independent experiments were expressed as mean ± SD. Student's t test was used for statistical analysis in two groups, and data from more than two groups were analyzed by one-way ANOVA. p< 0.05 was statistically significant.
